# Tailored Cytokine Optimization for *ex vivo* Culture Platforms Targeting the Expansion of Human Hematopoietic Stem/Progenitor Cells

**DOI:** 10.3389/fbioe.2020.573282

**Published:** 2020-09-25

**Authors:** André Branco, Sara Bucar, Jorge Moura-Sampaio, Carla Lilaia, Joaquim M. S. Cabral, Ana Fernandes-Platzgummer, Cláudia Lobato da Silva

**Affiliations:** ^1^Department of Bioengineering, Institute for Bioengineering and Biosciences, Instituto Superior Técnico, Universidade de Lisboa, Lisbon, Portugal; ^2^Hospital São Francisco Xavier, Centro Hospitalar de Lisboa Ocidental, Lisbon, Portugal

**Keywords:** *ex vivo* expansion, umbilical cord blood, human hematopoietic stem/progenitor cells, cytokines, process optimization

## Abstract

Umbilical cord blood (UCB) has been established as an alternative source for hematopoietic stem/progenitor cells (HSPC) for cell and gene therapies. Limited cell yields of UCB units have been tackled with the development of cytokine-based *ex vivo* expansion platforms. To improve the effectiveness of these platforms, namely targeting clinical approval, in this study, we optimized the cytokine cocktails in two clinically relevant expansion platforms for HSPC, a liquid suspension culture system (CS_HSPC) and a co-culture system with bone marrow derived mesenchymal stromal cells (BM MSC) (CS_HSPC/MSC). Using a methodology based on experimental design, three different cytokines [stem cell factor (SCF), fms-like tyrosine kinase 3 ligand (Flt-3L), and thrombopoietin (TPO)] were studied in both systems during a 7-day culture under serum-free conditions. Proliferation and colony-forming unit assays, as well as immunophenotypic analysis were performed. Five experimental outputs [fold increase (FI) of total nucleated cells (FI TNC), FI of CD34^+^ cells, FI of erythroid burst-forming unit (BFU-E), FI of colony-forming unit granulocyte-monocyte (CFU-GM), and FI of multilineage colony-forming unit (CFU-Mix)] were followed as target outputs of the optimization model. The novel optimized cocktails determined herein comprised concentrations of 64, 61, and 80 ng/mL (CS_HSPC) and 90, 82, and 77 ng/mL (CS_HSPC/MSC) for SCF, Flt-3L, and TPO, respectively. After cytokine optimization, CS_HSPC and CS_HSPC/MSC were directly compared as platforms. CS_HSPC/MSC outperformed the feeder-free system in 6 of 8 tested experimental measures, displaying superior capability toward increasing the number of hematopoietic cells while maintaining the expression of HSPC markers (i.e., CD34^+^ and CD34^+^CD90^+^) and multilineage differentiation potential. A tailored approach toward optimization has made it possible to individually maximize cytokine contribution in both studied platforms. Consequently, cocktail optimization has successfully led to an increase in the expansion platform performance, while allowing a rational side-by-side comparison among different platforms and enhancing our knowledge on the impact of cytokine supplementation on the HSPC expansion process.

## Introduction

Hematopoietic cell transplantation (HCT) continues to be the leading cell therapy for malignant and non-malignant blood-based disorders and advances in this field have expanded the options available for patients concerning graft source. Umbilical cord blood (UCB) is an accepted and appealing alternative source of hematopoietic stem/progenitor cells (HSPC) for HCT (Hough et al., 2016; Woolfrey et al., 2016). Compared with bone marrow (BM) or mobilized peripheral blood, UCB transplants have shown similar survival outcomes with lower chances of developing graft vs. host disease (GVHD) and lesser compatibility issues concerning human leukocyte antigen (HLA) matching (Rocha et al., 2001, 2004). However, low UCB volume recovered from births results in an unsatisfactory cell dose for transplants in adults, having initially limited transplants of a single UCB unit to pediatric patients (Wagner et al., 2014). In order to address this problem, *ex vivo* expansion of HSPC has been pursued. By manipulating UCB units to increase their cell yield, the drawbacks of single unit transplants (such as increased graft failure and delayed immune reconstitution) can potentially be surpassed (Kelly et al., 2009). Multiple strategies have been developed toward achieving a successful expansion, with several reaching phase III clinical trial level (Maung and Horwitz, 2019). Approaches have varied from promoting HSPC expansion with novel small molecules including StemRegenin-1 (Wagner et al., 2016), UM171 (Fares et al., 2014), and nicotinamide (Horwitz et al., 2014), co-culture with mesenchymal stromal cells (de Lima et al., 2012) and induction of Notch signaling pathways (Delaney et al., 2010).

Although different strategies have been explored, HSPC *ex vivo* expansion has always been largely based on the addition of exogenous cytokines (Lund et al., 2015). Numerous cytokines have been employed to promote HSPC expansion *ex vivo*, including fms-like tyrosine kinase 3 ligand (Flt-3L), granulocyte colony-stimulating factor (G-CSF), interleukin-3 (IL-3), interleukin-6 (IL-6), stem cell factor (SCF), and thrombopoietin (TPO) (Conneally et al., 1997; Ohmizono et al., 1997; Levac et al., 2005) (reviewed in Costa et al., 2018). However, selection of individual cytokines and their concentrations for an expansion cocktail has differed between existing strategies. Disparity of concentrations can reach 30-fold among similar cytokines included in different expansion protocols (Delaney et al., 2010; Çelebi et al., 2012). Whereas cytokine dosage may vary due to the nature of the expansion approach (e.g., targeted expansion of more primitive self-renewing hematopoietic stem cells compared to expansion of both hematopoietic stem cells and early committed progenitors), a defined and clear optimization rationale concerning cytokine supplementation has been lacking. Ignoring or underestimating optimization opportunities can have a negative impact on existing culture protocols, especially concerning cytokine supplementation. Suboptimal cytokine concentrations can cause underperformance of cell expansion driving misleading conclusions, especially when carrying out comparisons with other competitive strategies. On the other hand, overuse of cytokine supplementation has shown to interfere with HSPC self-renewal and promote unwanted differentiation (Zandstra et al., 1997a; Audet et al., 2002). Moreover, considering their significant cost, these abnormally high cytokine concentrations can also compromise process viability, cost-effectiveness and potential for clinical translation (Yoshida and Takagi, 2004; Aijaz et al., 2018). Thus, there is a clear gap in protocol standardization and optimization for current HSPC *ex vivo* expansion platforms.

With the lack of optimized platforms, current evaluation of the performance of various expansion approaches based on their published results might be inaccurate, since these platforms are most likely not performing at their peak production potential. Therefore, improper optimization of cytokine usage can affect decision-making and eventually be responsible for negligent or premature withdrawal of certain expansion approaches from the clinical approval pipeline. While improving existing expansion platforms, cytokine cocktail optimization will also enable a fair side-by-side comparison of current strategies.

Systematic studies on cytokine use in *ex vivo* expansion of HSPC will also support platforms toward an effective protocol for clinical applications based on good manufacturing practices (GMP). Besides highlighting the abovementioned cost reduction opportunities, cytokine optimization will also elucidate on important biological interactions between cytokines and cultured HSPC. The knowledge gathered from this relationship will benefit bioprocess engineering from a future manufacturing line perspective. The understanding of these cytokine requirements will have a direct impact on the feasibility of such a GMP-based expansion protocol, which is a priority for platforms at a clinical trial level (Kirouac and Zandstra, 2008). Although an initial focus on the cytokine cocktail existed during the early development of *ex vivo* HSPC expansion protocols, previous attempts to study cytokine influence are mostly based on simple dose-response studies and are outdated (Petzer et al., 1996; Ohmizono et al., 1997; Zandstra et al., 1997a,b, 1998; Ueda et al., 2000). Furthermore, due to major advances in *in vitro* culture of HSPC (e.g., development of serum-free medium formulations), tested culture conditions are inconsistent with expansion strategies presently in clinical trials, making the application of previous optimizations inadequate. With a considerable amount of HSPC expansion strategies in late-stage development, where major changes in the experimental procedure are rare, any cytokine optimization performed at this stage could endure. Thus, existing cytokine variation throughout current UCB-based expansion strategies was surveyed (reviewed in Kiernan et al., 2017; Costa et al., 2018). Despite some expected variants between strategies, we identified the trio of cytokines SCF, Flt-3L and TPO as the most used cytokine combination in the majority of expansion studies (reviewed in Costa et al., 2018), including those which have progressed to Phase I/II clinical trials (reviewed in Kiernan et al., 2017). By specifically selecting these three cytokines, we expect to boost the relevance of our study, turning its application more widespread.

Over the last years, we have gathered significant expertise in what concerns the *ex vivo* cultivation of human HSPC by establishing a co-culture system with BM MSC, in order to improve our understanding of the mechanisms underlying the hematopoietic supportive capacity of human MSC (da Silva et al., 2005, 2009, 2010; Gonçalves et al., 2006; Andrade et al., 2011, 2015). Having identified the aforementioned gap in the field, we tackled the issue with initial efforts focused on pursuing optimization of our established co-culture platform with BM MSC using statistical tools, such as design of experiments (Andrade et al., 2010). Unable to perform feeder-free HSPC expansion with the selected culture conditions, in particular for UCB cells (da Silva et al., 2005), our previous optimization study was restricted to a single expansion platform, exclusively performed in a co-culture system with BM MSC.

Using the same statistical approach based on experimental design, in the present study, we have determined unique optimal cytokine cocktails for two different HSPC expansions systems (i.e., HSPC expanded alone in a liquid culture system or co-cultured with a BM MSC feeder layer) currently exploited in clinical trials. By enhancing the cytokine contribution for each platform, we were able to level the field and perform a rational and pragmatic comparative study between both systems (liquid suspension culture vs. co-culture). By optimizing the established expansion platforms, we have reached a durable optimal cytokine cocktail to hopefully endure and facilitate the road to regulatory approval of a viable cell product based on expanded UCB-derived HSPC. Furthermore, by expanding HSPC from cryopreserved UCB MNC, we have made our optimization more reliable and applicable to the manufacturing scenario. Indeed, upon collection, UCB units are routinely kept cryopreserved in public/private banks worldwide. Also, we have shown that tailored cytokine optimization should be used as a tool to enable unbiased evaluation of existing strategies, rationally impacting the highly competitive field of *ex vivo* expansion of HSPC, namely (but not limited to) UCB-derived.

## Materials and Methods

### Human Tissues

Originally, UCB units and BM samples were obtained from healthy donors, upon informed consent, with the approval of the ethics committee of Hospital São Francisco Xavier, Centro Hospitalar de Lisboa Ocidental and of Instituto Português de Oncologia Francisco Gentil, Lisbon, Portugal, respectively (Directive 2004/23/EC of the European Parliament and of the Council of March 31st, 2004 regarding standards of quality and safety for the donation, procurement, testing, processing, preservation, storage and distribution of human tissues and cells, represented by the corresponding Portuguese Law 22/2007).

### Cell Isolation

Mononuclear cells (MNC) were isolated from UCB using a Ficoll [GE Healthcare, United States of America (United States), Supplementary Table 2] density gradient and washed with Phosphate Buffered Saline (PBS) (Sigma-Aldrich, United States) supplemented with 2 mM ethylenediamine tetraacetic acid (EDTA) (Sigma-Aldrich). MNC were incubated with an ammonium chloride solution (155 mM) (Sigma-Aldrich) to remove any possible contamination with erythrocytes. Purified MNC were cryopreserved in Recovery^TM^ Cell Culture Freezing Medium (Thermo Fisher Scientific, United States) and stored in a liquid/vapor phase nitrogen tank.

### CD34^+^ Cell Enrichment From UCB MNC

Cryopreserved UCB MNC were thawed in low glucose Dulbecco’s Modified Eagle’s Medium (DMEM) (Thermo Fisher Scientific) containing 20% fetal bovine serum (FBS) (Thermo Fisher Scientific) and 1% Antibiotic-Antimycotic (Thermo Fisher Scientific) (DMEM-20%FBS) supplemented with 10 μg/mL DNase I (Sigma-Aldrich). Cells were washed with MACS buffer and CD34^+^ cell selection was performed using a Human CD34 MicroBead Kit UltraPure (Miltenyi Biotec, Germany), following manufacturer’s instructions.

### Preparation of a BM MSC Feeder Layer

Bone marrow derived mesenchymal stromal cells were obtained from the Stem Cell Engineering Research Group (SCERG) cell bank, at the Institute for Bioengineering and Biosciences, Instituto Superior Técnico, Lisboa, Portugal. Cell isolation, expansion, characterization and preservation were done using previously established protocols (dos Santos et al., 2010). Cells from a single BM donor were used to establish feeder layers, mimicking an allogeneic universal donor. To establish the feeder layer, cryopreserved BM MSC, between passages P2-P5, were thawed in DMEM-20% FBS and plated at a cell density of 3000 cells/cm^2^ with low glucose DMEM supplemented with 10% FBS MSC-qualified (Thermo Fisher Scientific) and 1% Antibiotic-Antimycotic (DMEM-10%FBS). After cells reached confluency, growth arrest was promoted by treating cells during 2.5−3 h at 37°C and 5% CO_2_ with low glucose DMEM-10% FBS supplemented with 0.5 μg/mL of Mitomycin C (Sigma-Aldrich). Growth-arrested feeder layers were carefully washed twice with DMEM-10% FBS and incubated with the same medium until further use in co-culture experiments.

### *Ex vivo* Expansion of HSPC

Umbilical cord blood-derived CD34^+^ enriched cells were seeded on a 12-well plate at 30 000 cells/mL, using 2 mL of StemSpan^TM^ Serum-free Expansion Medium II (StemCell Technologies, Canada) per well (60 000 cells/well) supplemented with 1% Antibiotic-Antimycotic and defined cytokine cocktails composed of SCF, Flt-3L, and TPO (PeproTech, United States), with concentrations ranging between 0 and 100 ng/mL. Basic fibroblast growth factor (bFGF) (PeproTech) was additionally used in all conditions at a concentration of 5 ng/mL to support BM feeder layer cells during the co-culture experiments (Andrade et al., 2010). HSPC expansion was performed during 7 days at 37°C and 5% CO_2_. For co-culture with BM MSC, DMEM-10% FBS culture medium was removed from the growth arrested feeder layers and CD34^+^ enriched cells were carefully seeded on top as described above.

### Proliferative and *in vitro* Clonogenic Assays

#### Proliferation Assay

At day 7 of expansion, suspended and adhered HSPC (in the case of co-cultures) were harvested through forced pipetting. Cell number was determined using the Trypan Blue (Thermo Fisher Scientific) exclusion method. Fold increase in total nucleated cell number (FI TNC) was calculated by dividing the cell number at day 7 by the initial seeding cell number (day 0).

#### Colony-Forming Unit (CFU) Assay

Cells were characterized according to their capacity, as progenitor cells, to proliferate and differentiate into several hematopoietic lineages. Cells were resuspended in MethoCult^TM^ methylcellulose-based medium (STEMCELL Technologies) and seeded on a 24-well plate in triplicates. Colony formation was allowed for 14 days at 37°C and 5% CO_2_. Formed colonies [erythroid burst-forming unit (BFU-E), colony forming unit granulocyte-monocyte (CFU-GM), and multilineage colony forming unit (CFU-Mix)] were classified and counted by visual inspection using bright-field microscopy (Olympus CK40, Japan). Colony number was normalized by the number of seeded cells and multiplied by the TNC number. FI in the number of colonies was calculated by dividing the total colony number at day 7 by the respective of day 0.

#### Cobblestone Area Forming-Cells (CAFC)

A growth-arrested feeder layer of a murine stromal cell line (MS-5) was prepared similarly as previously described for BM MSC (Andrade et al., 2010). To further characterize their stemness, expanded and non-expanded HSPC were resuspended in MyeloCult^TM^ medium (STEMCELL Technologies) supplemented with 350 ng/mL of hydrocortisone (STEMCELL Technologies). Cells were seeded on a 24 well-plate in duplicates at 2000 cells/well and incubated for 14 days at 37°C and 5% CO_2_. Wells were visually inspected using a phase-contrast microscope (Leica DMI3000 B, Germany) for the presence of colonies of more than 5 cells with cobblestone-like morphology (phase-dim, compact grouped, angular shaped cells, Denning-Kendall et al., 2003) that were able to migrate beneath the murine feeder layer. Colony number was normalized by the number of seeded cells and multiplied by the TNC number. FI values relative to the number of CAFC colonies were obtained by dividing the CAFC colonies at day 7 by initial CAFC colony population at day 0.

### Cell Immunophenotype

Hematopoietic stem/progenitor cells phenotype was characterized by flow cytometry on a FACSCalibur^TM^ flow cytometer (BD Biosciences, United States). Briefly, harvested cells were washed with PBS, viability was assessed through a Far Red LIVE/DEAD^TM^ Fixable Dead Cell Stain Kit (Thermo Fisher Scientific), and cells were surface stained with previously titrated CD34 FITC (BioLegend, United States), CD34 PE (BioLegend), CD34 PerCP-Cy5.5 (BD Biosciences), and CD90 PE (BioLegend) at room temperature (RT) for 15 min. After PBS washing, cells were fixed in 1% formaldehyde (Sigma-Aldrich) at RT for 15 min. Data was analyzed using FlowJo v10 software (FlowJo LLC, United States).

### Cytokine Experimental Design

Response surface methodology was applied to optimize cytokine concentrations for the *ex vivo* expansion of HSPC (Box et al., 1978). A face-centered central composite (CCF) design was used to select concentrations for three different cytokines (SCF, Flt-3L, and TPO), resulting in 17 experimental points. The tested observational window was limited by a minimum concentration of 0 ng/mL and a maximum of 100 ng/mL, for every cytokine. With defined limits, concentrations were coded to simplify listing of experimental points [lower level (−1) − 0 ng/mL; center level (0) − 50 ng/mL; higher level (1) − 100 ng/mL]. The experimental points included three center points in order to gain an estimation of the experimental error. Effect on cytokine variation on FI TNC, FI CD34^+^ cells, FI BFU-E, FI CFU-GM, and FI CFU-Mix was investigated. These outputs were termed response variables (Y_n_). Every response variable was measured in a blinded manner, eliminating experimental bias. A second-order polynomial function was suggested to describe and model the experimental data.

(1) $$Y_{n}=K+\beta_{1}\,\left[X_{1}\right]+\beta_{2}\,\left[X_{2}\right]+\beta_{3}\,\left[X_{3}\right]+\beta_{12}\,\left[X_{1}\right]\,\left[X_{2}\right]+\beta_{13}\,\left[X_{1}\right]\,\left[X_{3}\right]+\beta_{23}\,\left[X_{2}\right]\,\left[X_{3}\right]+\beta_{11}\,{\left[X_{1}\right]}^{2}+\beta_{22}\,{\left[X_{2}\right]}^{2}+\beta_{33}\,{\left[X_{3}\right]}^{2}$$

Proposed second-order polynomial function as a behavior function for a specific response variable (Y_n_), considering three cytokines (X_1_, X_2_, and X_3_). This model includes an intercept (K), responsible for describing the response variable when no cytokines are present, and three different types of cytokine effects. These include main individual cytokine impact (β_i_ parameters), interaction between the different cytokines (β_i j_ parameters), and molecular effects within the same cytokine (β_i i_ parameters).

### Validation

Determined regressions were validated by comparing predicted values for each response variable with corresponding experimental values of cytokine combinations not included in the original concentration panel. Two different cytokine combinations, the optimized cytokine cocktail and a previously established cocktail (Z9; [SCF] = 60 ng/mL; [Flt-3L] = 55 ng/mL; [TPO] = 50 ng/mL) from our previous study (Andrade et al., 2010) were chosen to test the applicability of the regressions in its defined experimental design space.

### Statistical Analysis

Function fitting was performed using a backward stepwise regression. Briefly, all terms were considered in the regression. An iterative *F*-test on the overall regression was applied. In each step, when the respective *p*-Value was above the stipulated threshold (0.05), the least significant parameter was eliminated from the model. This was done repeatedly until the regression itself gained significance. Goodness of fit variables R-squared, Adjusted R-squared, and root mean squared error (RMSE) were determined to assess regression quality. Unless stated otherwise, plotted error bars and error intervals represent the standard error of the mean (SEM).

## Results

**FIGURE 1:**
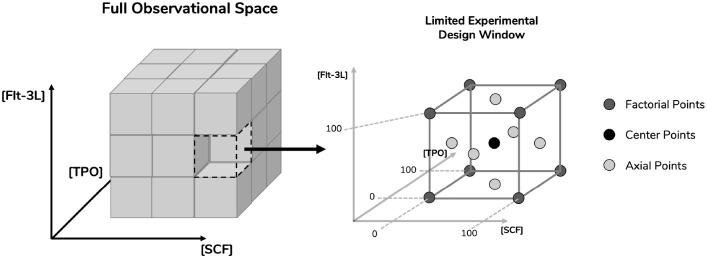
Definition of experimental design space for the optimization studies. Surface response methodology requires limitation of parameters in order to study response variables. Concentrations of the cytokines stem cell factor (SCF), fms-related tyrosine kinase 3 ligand (Flt-3L) and thrombopoietin (TPO) were selected as parameters. A limited experimental design window was selected from their full observational space with respective concentration ranges between 0 and 100 ng/mL. By incorporating three levels of dimensionality, the design space gained a cubic geometry. Having defined the design space, a face-centered central composite design was applied, which provided the experimental points necessary in order to reach the response surface. These include center points, axial points (located in the center of the cubic planes) and factorial points (located in the cubic vertices).

**FIGURE 2:**
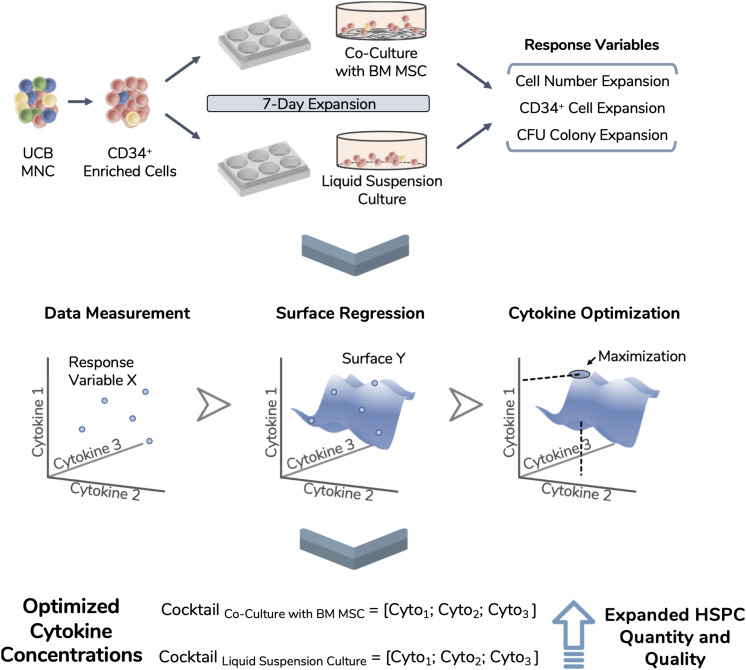
Experimental workflow of the performed optimization. Umbilical cord blood (UCB) mononuclear cells (MNC) were thawed and enriched for CD34 expression. These isolated cells were used as the starting population in two different expansion systems (i.e., liquid suspension culture and co-culture with bone marrow-derived mesenchymal stromal cells) and expanded during 7 days. Total nucleated cell number, CD34 expressing cell number and CFU readouts were selected for optimization and termed as response variables. Using an experimental design approach, 17 different cytokine combinations were used during expansion runs and response variables were tracked. Experimental data points were modeled, giving rise to unique response surfaces for each expansion system. By locating the surface maximum, each response variable originated an optimized cytokine cocktail, improving the quantity and quality of the expanded cell product.

Combinations of selected cytokines (SCF, Flt-3L, and TPO) were defined using an experimental design approach (Figure 1). A CCF design delineated a panel of 17 cytokine combinations, which included three repeated center points to assess intra-donor variability of UCB cells (Table 1). Cytokine concentrations were limited to an experimental design window between 0 and 100 ng/mL. FI TNC, FI CD34^+^, FI BFU-E, FI CFU-GM, and FI CFU-Mix were chosen as response variables for this optimization study, acting as measures of cytokine performance. Two different expansion platforms were studied, with HSPC being expanded alone in a liquid culture system (*CS_HSCP*) or co-cultured with a BM MSC feeder layer (*CS_HSPC/MSC*). Response variables were modeled and corresponding regression surfaces were maximized in order to uncover optimized cytokine cocktails for both expansion systems (Figure 2). Three independent donors of UCB cells were studied to include biological variability in the model. UCB CD34^+^-enriched cells (purity: [82–98%]) were expanded in serum-free conditions for 7 days using both expansion strategies.

**TABLE 1:** List of cytokine combinations derived from the face-centered central composite (CCF) design.

**Cytokine**	**Factorial points**								**Center points**			**Axial points**					
SCF	+	+	+	+	−	−	−	−	0	0	0	0	0	+	−	0	0
Flt-3L	+	+	−	−	+	+	−	−	0	0	0	0	0	0	0	+	−
TPO	+	−	+	−	+	−	+	−	0	0	0	+	−	0	0	0	0
*Combination*	*1*	*2*	*3*	*4*	*5*	*6*	*7*	*8*	*9*	*10*	*11*	*12*	*13*	*14*	*15*	*16*	*17*

*Concentrations values were symbol coded to facilitate identification and combinations were numbered to aid with cocktail recognition. In total, 17 combinations were defined, consisting of 8 factorial points, three repeated center points and 6 axial points. (+) 100 ng/mL; (0) 50 ng/mL; (−) 0 ng/mL.*

### Response Variable Measurement

**FIGURE 3:**
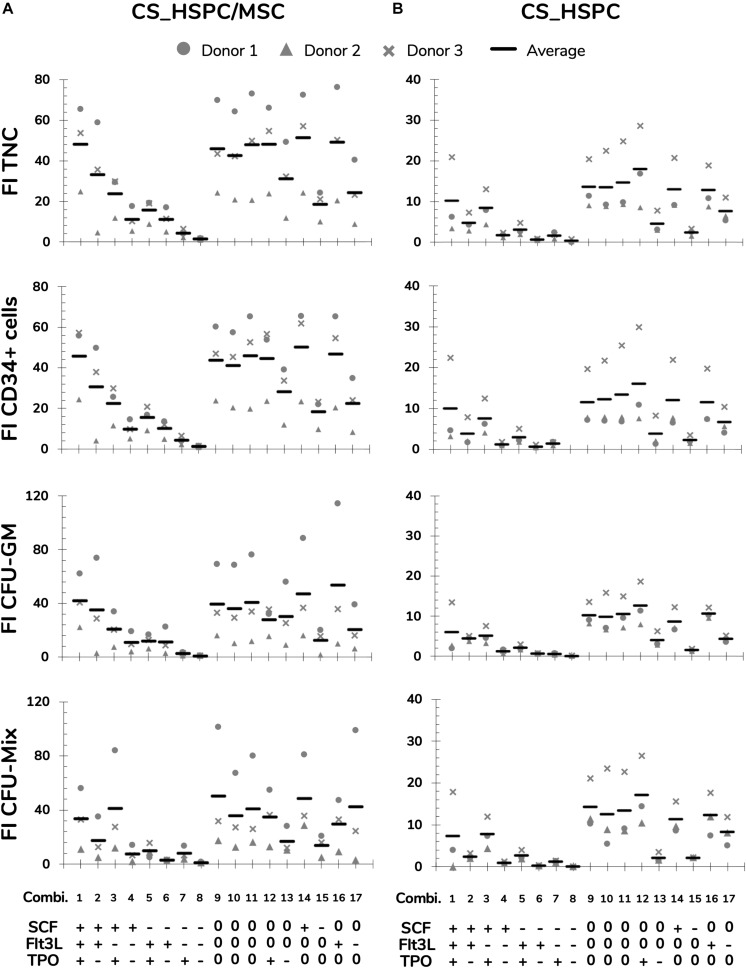
Measurements of response variables for two different expansions systems, HSPC suspension culture (CS_HSPC) and co-culture with bone marrow mesenchymal stromal cells (CS_HSPC/MSC). Throughout the entire cytokine panel of 17 combinations, values of fold increase (FI) of total nucleated cells (TNC), FI of CD34^+^ expressing cells, FI of colony-forming unit granulocyte-monocyte (CFU-GM) and FI of multilineage colony-forming unit (CFU-Mix) were followed. Cells isolated from three different donors were used for testing the response variables for CS_HSPC/MSC **(A)** and CS_HSPC **(B)**. (+) 100 ng/mL; (0) 50 ng/mL; (–) 0 ng/mL.

Selected response variables were successfully measured for three independent UCB unit donors. FI TNC number fluctuated considerably when expanding cells with the different cytokine combinations of the established panel in both expansion culture systems (coefficient of variation CV_CS_HSPC/MSC_ = 60 ± 3%; CV_CS_HSPC_ = 76 ± 1%; Figure 3). Taking into account every center point replicate, their low deviation (CV_Center_ = 13 ± 5%) demonstrated reproducibility of the expansion performance, discarding possible experimental error interference. Combinations with an absence of a certain cytokine caused a significant decrease in expansion capabilities, demonstrating the individual importance of the tested cytokines (Figure 3). Overall, cell expansion capacity varied to a higher extent at a lower range (0–50 ng/mL), while displaying similar culture performance for combinations with concentrations in the higher testing range (50–100 ng/ml). Nevertheless, cytokine panel screening resulted in a FI TNC reaction fingerprint that was coherent between donors. Although biological variability was present and the absolute values of measured variables were different, the overall pattern was very coincident. Additionally, this fingerprint was uniquely distinctive between expansion approaches.

The CFU assay contributed with three response variables (Figure 3 and Supplementary Table 1). Since BFU-E formation was always neglectable, it was not possible to progress with the variable FI BFU-E to the regression modeling phase. Without quantifiable BFU-E populations, CFU-GM and CFU-Mix were mirrored in their population percentage in each assay. FI CFU-GM and FI CFU-Mix demonstrated similar sensitivity to cytokine concentration variation as with FI TNC, but they developed their own cytokine fingerprint.

In contrast with the remaining response variables, CD34 expression did not show the same sensitivity toward different cytokine concentrations (not shown). Since CD34 expression exhibited minor influences by the cytokine panel, its respective response variable (FI CD34^+^ cells) revealed the same response pattern as the FI TNC number (Figure 3).

### Regression Determination and Analysis

**FIGURE 4:**
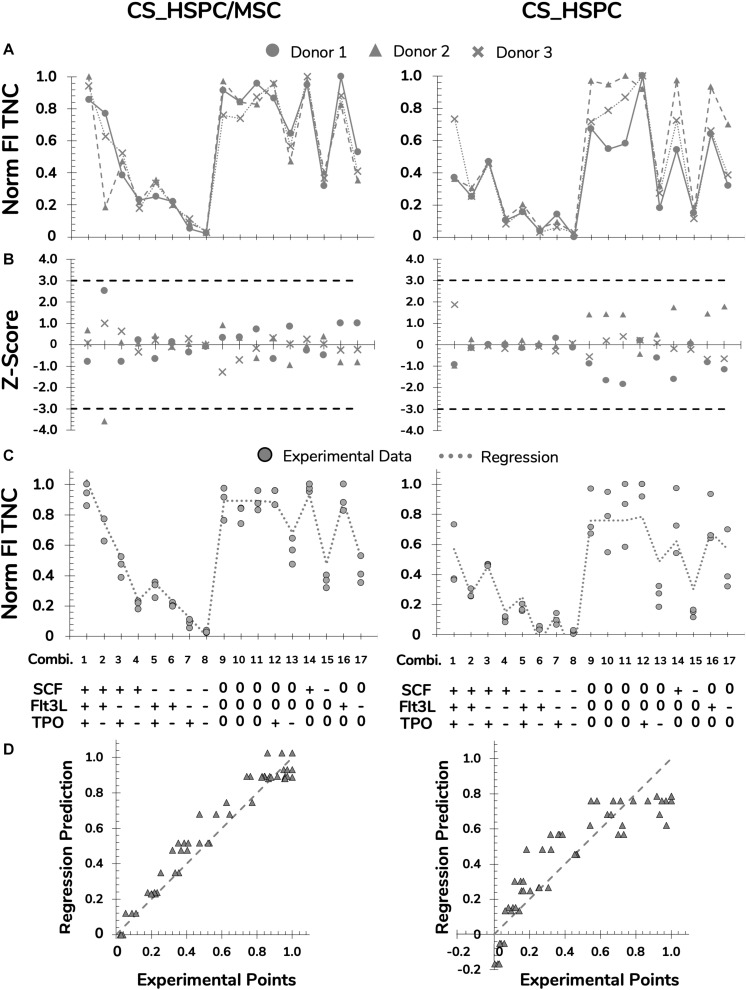
Preparation and polishing of experimental data with assessment of regression quality for FI TNC for both expansion systems, HSPC suspension culture (CS_HSPC) and HSPC co-cultured with BM MSC (CS_HSPC/MSC). **(A)** Data from cells retrieved from every UCB donor was normalized revealing coinciding reaction patterns, highlighting variability exclusively due to different cytokine combinations. **(B)** Outlier screening was performed through Z-score determination. Data points with absolute score values higher than 3 were labeled outliers and were consequently removed from their data set before proceeding to the regression determination. **(C)** After regression determination, experimental data points were compared with calculated regression. **(D)** Deviations between data points and regressions were visualized. Norm, normalized.

Several steps were taken to prepare and polish the response variables for regression modeling. Data from each donor was normalized to remove the biological variability on cell expansion intensity, highlighting the effects driven by the cytokine panel (Figure 4A and Supplementary Figures 1A, 2A, 3A). Prior to regression determination, outliers were detected through a Z-score method and eliminated (Figure 4B and Supplementary Figures 1B, 2B, 3B). Regressions for each response variable were calculated, reaching significance in every case (Table 2).

**TABLE 2:** Parameter estimations after regression determination and quality measures for each response variables and expansion system.

	**Normalized FI TNC**		**Normalized FI CD34^+^ Cells**		**Normalized FI CFU-GM**		**Normalized FI CFU-Mix**	
**Parameter**	**Estimate**	***p*-Value**	**Estimate**	***p*-Value**	**Estimate**	***p*-Value**	**Estimate**	***p*-Value**
**CS_HSPC/MSC**								
K	−	−	−	−	−	−	−	−
SCF	9.90 × 10^−3^	1.05 × 10^−9^	9.27 × 10^−3^	1.04 × 10^–8^	1.00 × 10^−2^	2.02 × 10^−5^	3.82 × 10^−3^	6.09 × 10^−6^
Flt-3L	9.89 × 10^−3^	4.62 × 10^−10^	9.95 × 10^−3^	8.47 × 10^−10^	6.88 × 10^−3^	2.05 × 10^−3^	6.70 × 10^−3^	1.90 × 10^−2^
TPO	5.67 × 10^−3^	3.35 × 10^−5^	6.66 × 10^−3^	4.18 × 10^−6^	1.09 × 10^−3^	4.98 × 10^−2^	1.24 × 10^−2^	5.40 × 10^−5^
SCF × Flt-3L	2.80 × 10^−5^	1.60 × 10^−4^	2.82 × 10^−5^	2.15 × 10^−4^	3.10 × 10^−5^	1.48 × 10^−2^	−	−
Flt-3L × TPO	−	−	−	−	−	−	−	−
SCF × TPO	1.60 × 10^−5^	2.23 × 10^−2^	1.51 × 10^5^	3.49 × 10^−2^	−	−	−	−
SCF^2^	−7.53 × 10(^5^	5.49 × 10^−8^	−6.95 × 10^−5^	5.23 × 10^−7^	−7.16 × 10^−5^	6.17 × 10^−4^	−	−
Flt-3L^2^	−7.60 × 10^−5^	4.53 × 10^−8^	−7.62 × 10^−5^	8.10 × 10^−8^	−4.89 × 10^−5^	1.53 × 10^−2^	−6.08 × 10^−5^	2.70 × 10^−2^
TPO^2^	−4.46 × 10^−5^	3.19 × 10^−4^	−5.35 × 10^−5^	4.35 × 10^−5^	−	−	−9.48 × 10^−5^	8.89 × 10^−4^
**Regression quality**								
R-squared (R^2^)	0.95		0.95		0.79		0.65	
Root Mean Squared Error (RMSE)	0.08		0.08		0.15		0.20	
Adjusted R-squared	0.94		0.94		0.76		0.60	
F-statistic vs. constant model	97.8		92.8		26.8		16.0	
*p*-Value	3.23 × 10^−24^		8.77 × 10^−24^		4.89 × 10^−13^		5.87 × 10^−9^	
**CS_HSPC**								
K	−1.65 × 10^−1^	9.90 × 10^−3^	−1.86 × 10^−1^	4.06 × 10^−3^	−1.84 × 10^−1^	5.52 × 10^−3^	−1.38 × 10^−1^	3.66 × 10^−2^
SCF	1.51 × 10^−2^	1.10 × 10^−7^	1.47 × 10^−2^	1.98 × 10^−7^	1.80 × 10^−2^	1.75 × 10^−10^	1.63 × 10^−2^	1.11 × 10^−7^
Flt-3L	6.55 × 10^−3^	8.85 × 10^−3^	6.30 × 10^−3^	1.14 × 10^−2^	8.56 × 10^−3^	3.05 × 10^−4^	−	−
TPO	8.02 × 10^−3^	1.65 × 10^−3^	9.31 × 10^−3^	3.26 × 10^−4^	2.37 × 10^−3^	2.09 × 10^−4^	1.15 × 10^−2^	5.53 × 10^−5^
SCF × Flt-3L	−	−	−	−	−	−	−	−
Flt-3L × TPO	−	−	−	−	−	−	−	−
SCF × TPO	−	−	−	−	−	−	−	−
SCF^2^	−1.19 × 10^−4^	5.67 × 10^−6^	−1.15 × 10^−4^	1.02 × 10^−5^	−1.49 × 10^−4^	7.78 × 10^−9^	−1.38 × 10^−4^	1.55 × 10^−6^
Flt-3L^2^	−5.41 × 10^−5^	2.40 × 10^−2^	−5.12 × 10^−5^	3.21 × 10^−2^	−6.57 × 10^−5^	3.04 × 10^−3^	−	−
TPO^2^	−5.01 × 10^−5^	3.59 × 10^−2^	−5.85 × 10^−5^	1.50 × 10^−2^	−	−	−8.22 × 10^−5^	1.86 × 10^−3^
**Regression quality**								
R-squared (R^2^)	0.78		0.79		0.78		0.71	
Root Mean Squared Error (RMSE)	0.16		0.16		0.16		0.19	
Adjusted R-squared	0.75		0.76		0.76		0.69	
F-statistic vs. constant model	26.4		27.9		31.5		28.1	
*p*-Value	4.51 × 10^−13^		1.77 × 10^−13^		1.68 × 10^−13^		9.96 × 10^−12^	

*Linear, interaction, and quadratic effects were included in an initial full quadratic model. A backward stepwise regression algorithm was used to correlate the experimental data with the proposed model. Significant estimations and their *p*-Values display the relationship between studied cytokines and the respective response variable. Quality of determined regressions and degree of correlations was expressed by the coefficient of determination (R^2^), root mean squared error (RMSE), adjusted coefficient of determination and statistic regression test and associated *p*-Value. Every regression and quality measures were determined for CS_HSPC/MSC and CS_HSPC.*

For both expansion systems, the hypothesized model was able to describe cytokine influence on the values of FI TNC to a considerable extent. For CS_HSPC/MSC, every projected term was significantly present, except for interaction effects between Flt-3L and TPO (Table 2). Negative quadratic effects were determined, leading to the existence of a concavity in the response surface and the existence of a local maximum in the tested range. On the other hand, for CS_HSPC, there were no interaction terms between cytokines. Also, regression fitting determined a negative intercept (*K* = −0.165), which has no biological translation and was disregarded.

Colony-forming unit assay response was modeled by a lesser number of significant parameters. FI CFU-GM and FI CFU-Mix had regressions with two particular characteristics. Unlike FI TNC number, some cytokines did not have a negative quadratic effect. Additionally, FI CFU-Mix for CS_HSPC showed total independence toward Flt-3L, lacking every type of cytokine effect considered in the model. In terms of overall regression quality, CFU-Mix originated fittings with lower quality (R${}_{\mathrm{CS}\,\_\,\mathrm{HSPC}/\mathrm{MSC}}^{2}$ = 0.65; R${}_{\text{CS\_HSPC}}^{2}$ = 0.71) compared to the remaining response variables.

As previously observed, FI CD34^+^ cells had similar behavior as the FI TNC number. Consequently, parameter estimation led to the same significant parameters and resembling values.

Regression performance was quantitatively assessed by the chosen quality measures. Although the regression quality varied, adjusted correlation coefficients maintained above 0.6 and were able to describe the experimental data significantly. Quantitative variables (FI TNC number and FI CD34^+^ cells) consistently produced higher quality regressions when compared to the semi-quantitative variables (FI CFU-GM and FI CFU-Mix) of the same expansion system. Quality assured regressions were then used to predict responses for the cytokine panel and were compared with experimental data points (Figure 4C and Supplementary Figures 1C, 2C, 3C). Residual determination was performed to visualize and quantify deviation between the model and data points (Figure 4D and Supplementary Figures 1D, 2D, 3D). With an average residual of 0.10 ± 0.02, CS_HSPC/MSC consistently showed increased correlation between the experimental data and the determined regressions for every response variable compared to the CS_HSPC with an average residual of 0.13 ± 0.01.

### Cytokine Concentration Optimization

**FIGURE 5:**
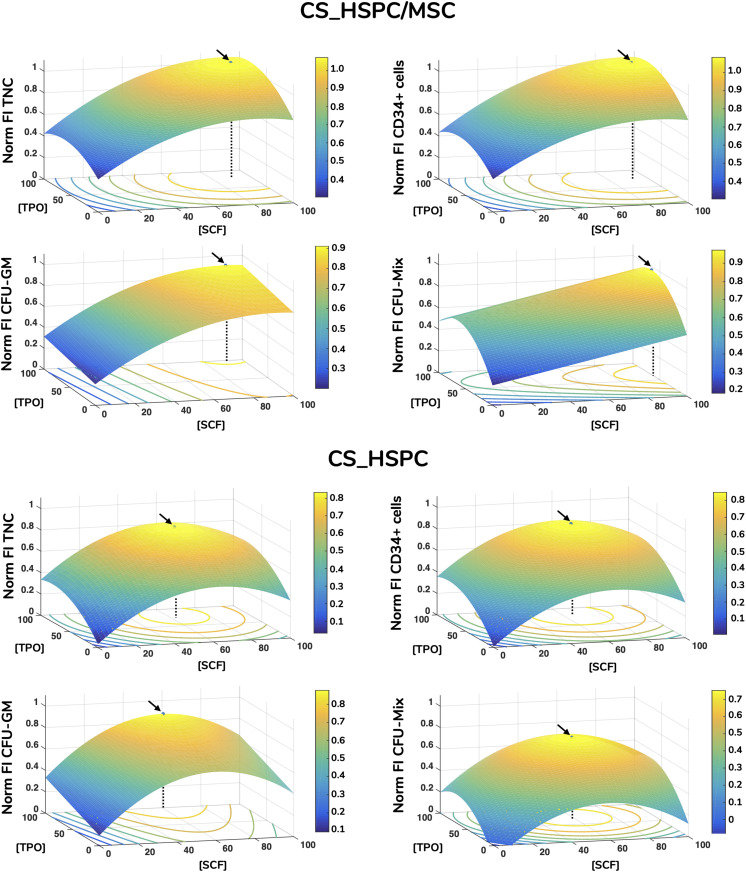
Response surface plots of every response variable with localization of optimal cytokine concentrations for HSPC suspension culture (CS_HSPC) and HSPC co-cultured with BM MSC (CS_HSPC/MSC). Calculated regressions were extrapolated to the whole design window, originating response surfaces. Surface plots containing the response surfaces were observed for the identification of a local optimal response. Regressions were maximized inside the limited design window, giving rise to the optimized cytokine cocktail. These are represented by a black arrow, while a dotted line highlights the corresponding cytokine concentrations that led to the maximum response. Flt-3L concentration was maintained constant at their respective optimal concentration. Norm – normalized.

**FIGURE 6:**
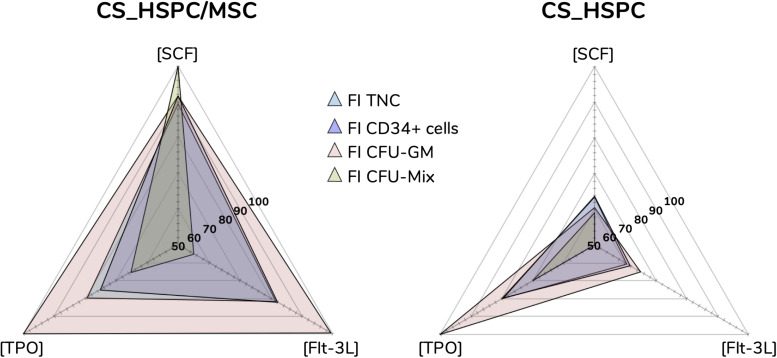
Optimal cytokine concentrations for every response variable and expansion system (ng/mL). Maximization of regressions led to optimal cytokine concentrations. Concentration plots displaying the different optimal cocktails observed for each response variable. Sharp differences were detected between both expansion systems, evidencing that cytokine influence is majorly dependent on the expansion approach.

Each calculated regression gave rise to a distinct response surface, limited by the design space. As predicted by the estimated parameters, every response variable produced a response surface with some degree of concavity, being a direct consequence of negative quadratic effects (Figure 5). Maximization of each surface inside the design window was performed. Concentrations corresponding to the maximum were defined as the optimal cytokine combination for that specific response variable (Table 3). Since the values of FI TNC and FI CD34^+^ cells possessed coinciding reaction fingerprints, their respective optimal combinations were very similar, which was observed for both expansion systems (Figure 6). Variables that did not possess negative quadratic effects for a certain cytokine in their regression caused their optimal concentration to reach the limit of the design space (100 ng/mL). Optimization was done for every response variable, which resulted in 4 optimized cytokine concentrations in each expansion approach. Due to their higher quality regressions and more quantitative nature, optimal concentrations of FI TNC number and FI CD34^+^ cells were given priority over the CFU output variables. Equal importance was given to the chosen variables and an average of their optimal combinations was performed to reach the final optimal combination (coined as AB20) for each expansion type.

**TABLE 3:** List of every optimized cocktail with respective denomination and selection of the final selected combination (ng/mL).

		**CS_HSPC/MSC**				**CS_HSPC**		
**Response variable**	**Cocktail**	**[SCF]**	**[Ftl-3L]**	**[TPO]**	**Cocktail**	**[SCF]**	**[Flt-3L]**	**[TPO]**
FI TNC	**HM1**	88	82	80	**H1**	63	60	80
FI CD34 + cells	**HM2**	92	82	75	**H2**	64	62	80
FI CFU-GM	**HM3**	92	99	100	**H3**	61	65	100
FI CFU-Mix	**HM4**	100	55	75	**H4**	59	−	70
Selected cocktail	**AB20**	90	82	77	**AB20**	64	61	80

*A total of 8 different optimal combinations were obtained. Prioritization for FI TNC and FI CD34^+^ cells with an average of their optimal concentrations, originated the selected cocktail for both expansion systems (AB20).*

### Validation

In order to validate the determined response surfaces for each response variable, their range of applicability was evaluated. Cocktails with concentrations not included in the initial experimental design panel are excellent candidates to assess predictive capabilities of calculated regressions. Besides the optimized cocktails (AB20), the combination of cytokines from our previous study (Z9) (Andrade et al., 2010), determined exclusively for the co-culture expansion system and using a different serum-free culture medium formulation, was also selected for validation studies ([SCF] = 60 ng/mL; [Flt-3L] = 55 ng/mL; [TPO] = 50 ng/mL).

**FIGURE 7:**
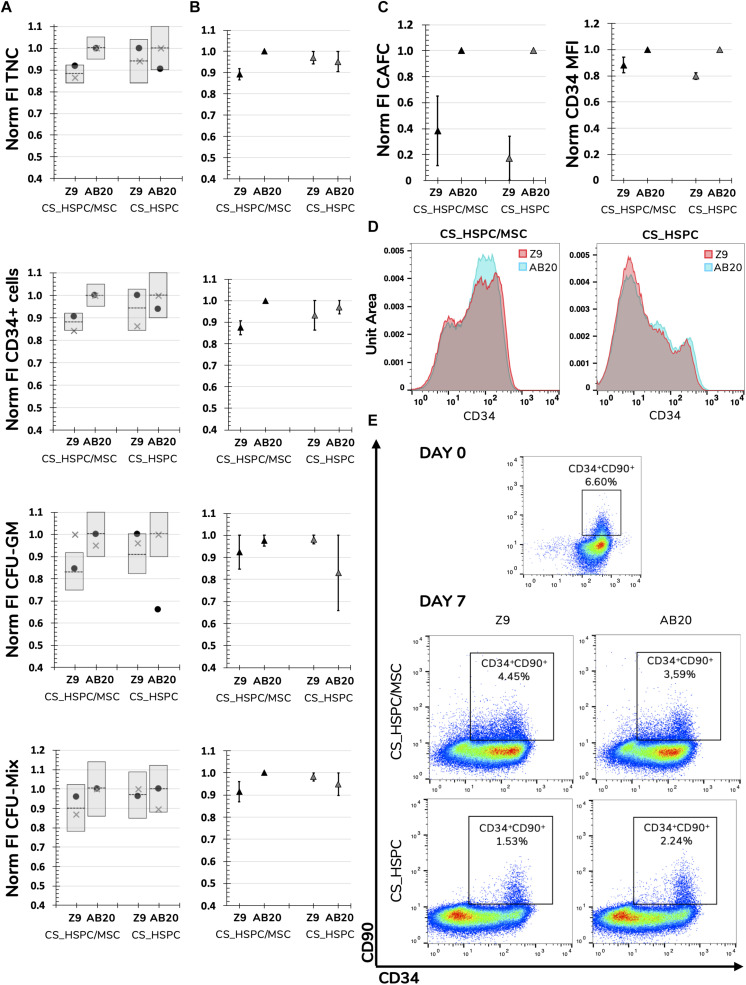
Validation of determined response surfaces and in-depth analysis of AB20 cocktails. **(A)** AB20 and Z9 cocktails were used as validation tests for calculated regressions. Predictability of regressions was analyzed by comparing function predictions and respective confidence intervals with experimental confirmation for every response variable and expansion system. Prediction represented by dashed line and confidence intervals by gray columns. **(B)** Average of two different donors showed that biological variability did not affect the predicted outcomes of comparison between Z9 and AB20. AB20 performed better or similar to Z9 cocktails as anticipated by the prediction and respective 95% confidence intervals. **(C)** Further comparison highlighted that benefits of AB20 cocktail determination went beyond selected response variables. Expansion using AB20 cocktails led to higher fold increase in CAFC and higher CD34 median fluorescence intensity. **(D)** Representative histogram of CD34 expression demonstrating that AB20 cocktails are able to delay loss of this marker during expansion. **(E)** Representative dot plots of CD34 and CD90 expression before and after expansion using both culture systems and cocktails. Initial CD34^+^CD90^+^ population is mostly lost during expansion, although a residual population percentage is observable in every condition. Mixed results were visible concerning maintenance of the more primitive population. Populations were previously gated for live cells using a viability assay. Data is represented by the mean ± standard error of the mean.

Respective regressions were applied to determine predicted responses of each variable. Also, confidence intervals were determined to define the expected range of prediction variation. HSPC expansion using the selected conditions was performed and resulting experimental data compared. Only 2 out of 32 experimental points (6.25%) were outside the predicted range, FI CFU-GM expanded with Z9 in CS_HSPC/MSC and FI CFU-GM expanded with AB20 in CS_HSPC (Figure 7A).

If regressions were to be used as a comparison tool and AB20 and Z9 considered as competitors, every regression would able to successfully predict the outcome between them. FI TNC number and FI CD34^+^ cells using a AB20 combination in CS_HSPC/MSC were considerably higher when compared to the Z9 cocktail (FI TNC_CS_HSPC/MSC_: 1.00 ± 0.00 vs. 0.89 ± 0.03; FI TNC_CS_HSPC_: 0.95 ± 0.05 vs. 0.97 ± 0.03) (FI CD34${}_{\mathrm{CS}\,\_\,\mathrm{HSPC}/\mathrm{MSC}}^{+}$: 1.00 ± 0.00 vs. 0.87 ± 0.03; FI CD34${}_{\text{CS\_HSPC}}^{+}$: 0.97 ± 0.03 vs. 0.93 ± 0.07) (Figure 7B). The remaining comparisons resulted in no substantial difference between tested cytokine cocktails, as predicted by their respective regressions. CD34 median intensity fluorescence (MFI), CAFC assays and levels of expansion of cells with a more primitive phenotype (%CD34^+^CD90^+^) were also analyzed to discern the effects of the AB20 combination on other relevant clinical variables (Figures 7C–E). Coherent increases of CD34 MFI in both culture systems using the AB20 cocktail were observed when compared to the Z9 cocktail (CD34 MFI_CS_HSPC/MSC_: 1.00 ± 0.00 vs. 0.88 ± 0.06; CD34 MFI_CS_HSPC_: 1.00 ± 0.00 vs. 0.80 ± 0.03). In terms of FI CAFC, AB20 cocktails in both expansion systems originated more colonies. Optimized cocktails were responsible for an increase of 2.5 ± 0.3 in CAFC compared to Z9 in the CS_HSPC/MSC, while AB20 also produced 4.7 ± 1.1 more colonies than Z9 in the CS_HSPC. On the other hand, AB20 cocktails had mixed performance concerning expansion of primitive progenitors. Both cocktails maintained a residual population percentage of CD34^+^CD90^+^ cells regardless of the culture system, with AB20 resulting in an average of 2.59 ± 0.68% and Z9 in an average of 2.65 ± 0.74%.

### Differential Cytokine Influence

**FIGURE 8:**
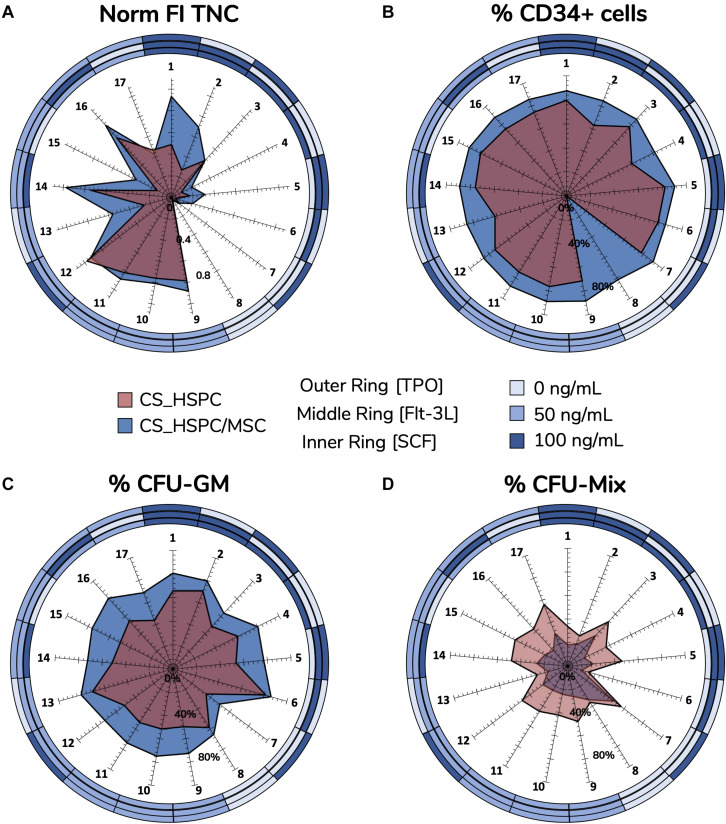
Reaction fingerprints obtained out of the 17 cytokine combinations. Information obtained from creating response surfaces can be exploited to further assess the relationship between an expansion system and cytokine use. Unique reaction fingerprints were determined for normalized FI TNC **(A)**, percentage of CD34^+^ cells **(B)**, percentage of CFU-GM **(C)**, and percentage of CFU-Mix **(D)**. Circular rings around plots display respective cytokine concentrations associated with each data point. CS_HSPC/MSC appear to synergize better with cytokines, except for CFU-Mix. Percentage of CD34 expression for cytokine combination 8 in the CS_HSPC was not quantified due to insufficient cell number.

This systematic method of achieving optimization provided considerable insight into the relationship between cytokines and cells in both expansion systems (i.e., HSPC culture with/without a BM MSC feeder layer). Cytokine reaction fingerprints were previously mentioned and were compared to highlight differences in cytokine influence (Figure 8).

Normalized FI TNC number (Figure 8A) displayed significative differences in several specific combinations, leading to three main observations. Firstly, the total fingerprint area for this variable for CS_HSPC/MSC was higher compared to the CS_HSPC (Area_CS_HSPC/MSC_ = 1.02 vs. Area_CS_HSPC_ = 0.59). Thus, the presence of a feeder layer appears to synergize with cytokine benefit during culture, boosting expansion levels closer toward their maximum performance. Moreover, the reaction fingerprints show that CS_HSPC is more vulnerable to the lack of an individual cytokine than CS_HSPC/MSC. Cells expanded in co-culture display an alleviated negative response whenever a combination without the presence of a cytokine was used. Lastly, adverse effects in cell expansion performance due to excess of cytokines are evident in CS_HSPC, shown by the transition between center points (combination 9, 10, and 11) and the combination with highest concentration of each cytokine (combination 1).

Lack of sensitivity of CD34 expression (Figure 8B) to the cytokine panel led to a circular-shaped reaction fingerprint. Over the entire cytokine panel, CS_HSPC/MSC displayed a reduced CV of 4.5 ± 3.9% for CD34^+^ cell percentage, while CS_HSPC exhibited a CV of 12.4 ± 8.8%. Nevertheless, CS_HSPC showed some dependence on TPO, since combinations without TPO had some negative impact on CD34 expression. Figure 8B evidences the impact of losing TPO for CS_HSPC. A decrease in CD34^+^ cell percentage of 16.4% was observed between combination 1 to 2, 17.3% from combination 3 to 4 and 12.5% from combination 12 to 13.

Colony-forming unit outputs (Figures 8C,D and Supplementary Table 1) had complementary reaction fingerprints, due to insignificant BFU-E quantification. Percentage of CFU-GM had low variation due to the cytokine panel, although some differences were visible. Fingerprint area was ubiquitously larger for co-culture system (Area_CS_HSPC/MSC_ = 17 142 vs. Area_CS_HSPC_ = 9838), demonstrating that its priming toward the granulocyte-macrophage lineage did not change with different cytokine cocktails. Clear benefits were apparent from Flt-3L supplementation, whereas TPO seemed to influence against CFU-GM development. This was more obvious in CS_HSPC, where combinations with those features (combination 2, 6, and 13) caused the differences in CFU-GM percentage between fingerprints of both systems to narrow.

### Comparison of Expansion Strategies

**FIGURE 9:**
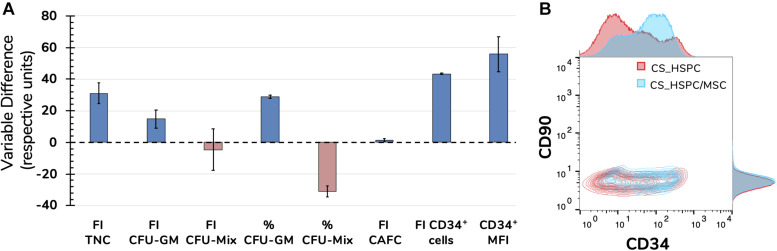
Side-by-side comparison between expansion systems with respective optimal cytokine concentrations (*n* = 2). **(A)** A number of significant variables concerning HSPC expansion were chosen as comparison criteria between CS_HSPC (red) and CS_HSPC/MSC (blue). Co-culture displayed superior performance in most variables, with the exception of FI CFU-Mix and percentage of CFU-Mix. **(B)** Contour plot of CD34 and CD90 expression after 7 days expansion with AB20 cocktail. At day 7, CS_HSPC/MSC demonstrated a substantially different CD34 expression profile, being able to retain expression of CD34 more effectively when compared to CS_HSPC. Data is represented by the mean ± standard error of the mean.

Upon completion of the optimization approach, cytokine contribution in each HSPC expansion system was maximized allowing a fair side-by-side comparison of the two studied expansion platforms. Several variables were followed during cell expansion at their maximum cytokine strength. The overall scale was clearly favorable toward the co-culture system, with six out of eight measures (75%) evidencing a better performance (Figure 9A). However, CS_HSPC demonstrated more capability toward promoting mixed colonies in the CFU assay in detriment to granulocyte-macrophage colonies. Although none were able to maintain the initial CD34 phenotype of UCB cells after 7 days, CS_HSPC/MSC showed it was able to significantly reduce the loss of this surface marker in cultured cells with a positive CD34 MFI difference compared to the CS_HSPC of 55.8 ± 7.8. When compared, the respective CD34 expression histograms appear almost mirrored (Figure 9B). Thus, with their cytokine cocktails optimized, expansion using a co-culture system demonstrated an overall superior potential in generating an expanded a HSPC product with higher retention of CD34 expression and primed for originating more CFU-GM.

## Discussion

With cell therapy manufacturing gaining traction as more advanced cell-based therapies get approved and reach the commercialization stage, efforts have been made in promoting the adoption of “Quality by Design” (QbD) guidelines, including process optimization and experimental design, while encouraging their implementation early on during the research and development phase (Rathore and Winkle, 2009; Lipsitz et al., 2016).

Experimental design of cytokine cocktails has been previously pursued, especially during initial studies on UCB-derived HSPC *ex vivo* expansion (Andrade et al., 2010; Pineault et al., 2011). However, applicability of the aforementioned studies to current expansion strategies is restricted. Whether due to having been performed in non-human cells (Audet et al., 2002), used to study *ex vivo* hematopoietic differentiation rather than HSPC expansion (Cortin et al., 2005; Lim et al., 2011) or to the lack of surface response models and optimization (Petzer et al., 1996; Tursky et al., 2012), previous attempts struggle in being transposed to present expansion protocols. This can be justified by a gradual improvement of HSPC expansion protocols, where innovation has eventually led to inconsistencies between culture conditions in current strategies and in abovementioned optimizations. Basal culture media have had their own dynamic evolution over time. Earlier culture media used in *ex vivo* HSPC expansion protocols were typically composed of basic formulations, having been developed for more generic cell culture applications. These usually required supplementation with FBS in order to enrich the formulation to allow for cell expansion, with such basal media varying between Minimum Essential Medium Eagle − Alpha Modification (α-MEM) (Amsellem et al., 2003; de Lima et al., 2008; Horwitz et al., 2014), Iscove’s modified Dulbecco’s Medium (IMDM) (Gilmore et al., 2000; Çelebi et al., 2012) and others (reviewed in Costa et al., 2018). With the development of culture media specifically for human HSPC expansion, aligned with growing concerns with the use of FBS as an undefined supplement, formulations were developed to be serum-free, with protocols implementing specialized culture media such as X-VIVO^TM^ 10 medium (Kögler et al., 1999), QBSF-60 serum-free medium (Qiu et al., 1999; Gonçalves et al., 2006), StemLine^®^ stem cell expansion medium (Tiwari et al., 2013) and StemSpan^TM^ serum-free expansion medium (Delaney et al., 2010; Fares et al., 2014; Wagner et al., 2016; Bari et al., 2018). This has benefited cell expansion results but ultimately compromised the applicability of previous optimizations described in the literature. Although we have contributed toward the resurgence of optimization of culture conditions targeting the expansion of human HSPC, our own initial study on cytokine supplementation optimization was performed with QBSF-60 medium as the established expansion medium (Andrade et al., 2010). This proprietary serum-free medium was originally designed to support human CD34^+^ cells (Qiu et al., 1999).

With StemSpan^TM^ (STEMCELL Technologies) medium being more prevalent in latest studies on HSPC *ex vivo* expansion and in most advanced clinical trials testing expanded HSPC (Wagner et al., 2016; Zonari et al., 2017; Bari et al., 2018; Calvanese et al., 2019; Cohen et al., 2019), we have seized this opportunity to achieve an enduring optimization with direct impact on ongoing late-stage development of cell therapies based on *ex vivo* expanded HSPC from UCB. However, the formulation StemSpan^TM^ serum-free medium used herein still has undisclosed supplements and some animal-derived components in its formulation (i.e., bovine albumin). In the absence of a clearly defined and disclosed formulation, possible extrapolation of our results to different media is limited and any animal-derived components increase the risks of contamination. Nevertheless, with the cell therapy manufacturing field pressing for animal-component free and chemically defined formulations, StemSpan^TM^ medium has currently adapted to those needs. If existing or novel strategies decide to transfer to such formulations through regulatory pressure or due to new GMP guidelines, our optimization study with StemSpan^TM^ stands a considerable chance in maintaining its applicability on clinical-grade *ex vivo* HSPC expansion.

In our work, we have aimed at achieving an enduring optimization of cytokine supplementation for two clinically relevant *ex vivo* HSPC expansion platforms, CS_HSCP and CS_HSPC/MSC (i.e., HSPC being expanded alone in a liquid culture system or co-cultured with a BM MSC feeder layer, respectively). With cytokines maintaining a significant role in protocols of *ex vivo* expansion of human HSPC (Lund et al., 2015), namely those from UCB, optimization of the used concentrations through experimental design is crucial. When attempted, optimization applied to biological issues with inherent variability is largely determined by the existence of a donor-independent pattern. Once approved as a therapy, UCB-derived expanded HSPC will hope to be produced from a single cord unit. Therefore, donor variability is a central issue and must be considered when performing such studies and thus selected response variables were followed for three different donors to increase the robustness of the optimized cocktail (Csaszar et al., 2013). As expected, biological variability was present and may be partly related to differences observed in the initial CD34 expression after enrichment (Andrade et al., 2011). Interestingly, this variability did not prevent the appearance of a recurring pattern in every response variable (Figure 4 and Supplementary Figures 1–3). Although different donors of UCB cells originated different absolute values for the selected response variables, cell-cytokine relationship did not change and cytokine optimization was carried out.

Early on, we observed that our response variables displayed greater fluctuations with lower concentrations of the defined cytokine panel (SCF, Flt-3L, and TPO). A certain degree of cytokine saturation was apparent from the center points (concentrations of 50 ng/mL) onward (Figure 3). With many expansion platforms being employed in clinical trials using concentrations of 100 ng/mL or higher (Boitano et al., 2010; Delaney et al., 2010; de Lima et al., 2012; Fares et al., 2014), there is an observable overuse of cytokine supplementation without a rational justification. Excess amount of these molecules will be responsible for unnecessary costs, which might jeopardize the implementation of the respective potential cell therapies (Csaszar et al., 2013). Eventually, this overload of cytokine molecules can also have a negative impact on the cells themselves, since it largely differs from the levels of cytokines that HSPC experience *in vivo*. With concentrations of cytokines in the BM ranging in the picomolar (Wodnar-Filipowicz et al., 1993; Huchet et al., 2003; Zhang et al., 2016), several groups may be crippling their expansions with HSPC overstimulation. Interestingly, in contrast to the four main response variables (FI TNC, FI CD34^+^ cells, FI CFU-GM, and FI CFU-Mix), CD34 expression observed post-expansion was an exception to the previously described behavior. This surface marker expressed by HSPC did not display any significant variation to the cytokine panel but did vary with other factors, such as cell expansion platform (CS_HSPC vs. CS_HSPC/MSC) and culture duration (Figure 8B). In general, selected response variables met optimization requirements, such as pattern emergence and fluctuation inside the design space.

As mentioned beforehand, modeling biological behavior with precision can be a challenge, depending on the nature of the selected response variables. Naturally, better quality regressions arose from more quantitative measures, such as FI TNC number (Table 2). Both CFU and CAFC outputs suffer from some subjectivity, inherent to these specific assays, requiring a significant dependence on experimental technique to originate results that make modeling possible (Purton and Scadden, 2007; Powell et al., 2016). The existence of a single outlier proves the quality of the response variable measurements.

Taking each of these regressions into account, CS_HSPC/MSC was able to produce more consistent results than its counterpart system (i.e., without a feeder layer). The presence of a BM MSC feeder layer (originally anticipated to better recreate the HSPC niche *ex vivo*) appears to create a buffer zone environment which is capable of making responses and expansion performance more uniform. This may be related with the specific cell expansion dynamics of a co-culture setting. During this type of culture, HSPC that adhere directly to the stromal feeder layer and are phase-bright (i.e., do not migrate underneath the feeder layer) become responsible for most of the cell division observed over the culture duration (Alakel et al., 2009). Although the fraction of non-adherent or suspended cells typically has the highest fold increase in cell number, these cells themselves do not seem to be proliferatively active. Only phase-bright adherent cells were observed to have an active cell cycle with a considerable cell number in the G2/M phase (Jing et al., 2010). Thus, attached HSPC, which saturate the entire stromal layer, are responsible for the increase in the non-adherent cell fraction observed by releasing their progeny into suspension (Jing et al., 2010). This behavior may justify lower cell expansion variation, since contact area saturation of the stromal layer appears to become the main regulator of proliferation, creating a stable cell expansion mechanism. Although co-culture introduces more biological factors, the results obtained for this platform have displayed reduced experimental deviation. Despite this, every response variable in both systems originated a good degree of correlation in their respective regressions, despite the existence of some expected variability.

Regression manipulation gave origin to a total of 8 optimal combinations, resulting from the selection of four response variables (FI TNC, FI CD34^+^ cells, FI CFU-GM, and FI CFU-Mix) for two different expansion systems (Figure 6). Prioritization of variables was required to appoint a single and representative optimal cytokine combination for each expansion platform. For most clinical trials related with *ex vivo* expansion of HSPC, TNC and number of CD34^+^ cells are selected as critical parameters (Wagner et al., 2016; Cohen et al., 2019). Considering their significant clinical relevance allied to their higher regression quality, these two measures (i.e., FI TNC and FI CD34^+^ cells) were chosen to define the optimal cocktails for each culture system. Following the described rationale, prioritization of variables led to determination of optimal cocktails (AB20) for the CS_HSPC ([SCF] = 90 g/mL; [Flt-3L] = 82 ng/mL; [TPO] = 77 ng/mL) and for the CS_HSPC/MSC ([SCF] = 64 ng/mL; [Flt-3L] = 61 ng/mL; [TPO] = 80 ng/mL) (Table 3). These cocktails were responsible for expansion results up to 49 FI TNC and 33 FI CD34^+^ cells for the CS_HSPC and 75 FI TNC and 70 FI CD34^+^ cells for the CS_HSPC/MSC from a single UCB unit. When comparing with clinical trial data using these types of platforms (Median FI TNC = 56, Median FI CD34^+^ cells = 4 for cytokine-based expansion, i.e., feeder free) and Median FI TNC = 12, Median FI CD34^+^ cells = 30 for co-culture expansion (Lund et al., 2015), cell expansions obtained herein with optimized cocktails demonstrated competitive outcomes, surpassing the performance of most reports of *ex vivo* HSPC expansion in similar platforms. Additionally, with the optimized cytokine cocktails displaying concentrations lower than many current protocols (e.g., Boitano et al., 2010; Delaney et al., 2010), we have highlighted avoidable costs and uncovered an opportunity for a cost-effectiveness measure. These results demonstrate the need for tailored optimization in improving the viability and financial feasibility of UCB-derived HSPC expansion platforms.

Following optimization, regression-derived response surfaces required validation in order to confirm their donor-independent applicability. Validation was successfully completed using two different cocktails, the determined optimal cocktails (AB20) and a previously established optimized cocktail by our group (Z9) (Andrade et al., 2010). Expansion outcome and behavior using these cocktails performed as projected by their respective regressions (Figure 7A). The few out-of-bounds experimental points were associated with the semi-quantitative nature of the CFU assay. When compared, AB20 cocktails outperformed or matched Z9 performance concerning the four response variables. Interestingly, AB20 cocktails were able to overtake Z9 in other important measures that were not included in the initial experimental design, including CD34 mean fluorescence intensity and CAFC formation (Figures 7C,D). However, since AB20 and Z9 cocktails were located in the higher concentration range, their comparison was challenging, since lower variations of response variables were previously highlighted for that range. As expected, with concentrations differences lower than 30 ng/mL, determined regressions predicted only slight differences between AB20 and Z9 for some response variables. Nevertheless, solid predictive capacity was demonstrated and optimized cocktails still showed superior expansion performance.

Throughout this study, we have once more confirmed the important role cytokines play in promoting HSPC expansion. Taking advantage of this optimization, we also focused on the cell-cytokine relationship to further complement our comparison between CS_HSPC and CS_HSPC/MSC. By identifying the existence of unique reaction fingerprints, we were able to shed light on the different impact cytokines have on both studied expansion platforms. These fingerprints showed obvious distinctions in cytokine reaction behavior. In agreement with previous observations, the BM MSC feeder layer seems to develop a protective microenvironment around the HSPCs, resembling their role in the BM niche. In fact, FI TNC reaction fingerprint from CS_HSPC evidenced its higher vulnerability to early culture saturation by excess quantities of cytokines (Figure 8A). Indeed, the presence of a BM MSC feeder layer was able to ameliorate negative cytokine inhibition. When adding an interactive feeder layer, the network of individual and synergistic cytokine effects changes and gains complexity (Kirouac and Zandstra, 2006). While the exogenous cytokines added to the culture medium in both systems are the same, the environment of endogenous cytokines and their respective quantities change due to feeder cell presence. With a very dynamic secretome, MSC are known to produce other cytokines that promote HSPC expansion (Lund et al., 2015). By better mimicking the hematopoietic niche with this stromal component, the microenvironment is able to reach a higher number and level of synergies which can potentially lead to higher cell expansion yields (Wagner et al., 2007; Méndez-Ferrer et al., 2010).

Knowledge from these reaction fingerprints and their regressions may be used for purposes other than the expansion of UCB-derived HSPC for HCT. Revived interest in autologous gene therapy has consolidated the application of expanded adult HSPC for treatment of genetic hematological diseases (Naldini, 2015; Dunbar et al., 2018). Approval of Strimvelis, a gene therapy product of transduced autologous BM-derived CD34^+^ cells for treatment of severe combined immunodeficiency due to adenosine deaminase deficiency, was a milestone in the field and represents the considerable potential that expanded HSPC have in gene therapy (Hoggatt, 2016). Other areas within the hematological field can also potentially take advantage of these regression strategies to tailor HSPC culture for their own needs. There has been interest in using UCB-derived HSPC culture platforms for the differentiation of cells toward the lymphoid lineage for use in immunotherapy (e.g., donor lymphocyte infusions, tumor-infiltrating lymphocytes, etc., Singh and Zúñiga-Pflücker, 2018). Both culture systems included in this study have been explored for such purposes. CS_HSPC/MSC has been shown to have potential as a system to maintain early lymphoid progenitors (i.e., CD34^+^CD7^+^ cells) (da Silva et al., 2010) and support the generation of functional natural killer and dendritic cells (Frias et al., 2008). On the other hand, CS_HSPC in combination with the small molecule StemRegenin-1 has been recently used for generation of progenitor T cells (Singh et al., 2019). Exploitation of these expansion systems for such different applications can also largely benefit from the cytokine optimization strategy established in the present study and the information gathered on the effects of cytokines on cultured cells.

To our knowledge, cytokine optimization has not been used as a tool to enable a correct side-by-side comparison of different strategies. This evaluation is critical for decision-making over which platform should be supported for clinical trial progression or apply for regulatory approval. Criteria for the selection of cytokine concentrations have roughly been the same throughout different types of expansion culture systems, ignoring high specificity of each strategy. Without determining unique cytokine cocktails for each one, direct comparison of published results in an unstandardized manner may cause unrealistic conclusions. By pursuing a tailored cytokine optimization in two expansion approaches, these may be rightfully compared at their full cytokine potential, making their critical steps and parameters more easily identifiable (Lipsitz et al., 2016).

In the optimized conditions of our study, CS_HSPC/MSC undoubtedly showed better capabilities in promoting HSPC expansion, which explains the progression of *ex vivo* mesenchymal-cell coculture through the clinical trial pipeline (de Lima et al., 2012). In our assessment, CS_HSPC/MSC proved to have a superior production capacity as a platform concerning every studied variable except for FI CFU-Mix (Figure 9). Notably, CD34 expression, which displayed reduced variability due to cytokine effects, was observed to be consistently higher in an HSPC/MSC co-culture setting. This difference was originally observed by comparing cytokine reaction fingerprints of the percentage of CD34^+^ cells (Figure 8B) and was quantitively confirmed in optimal conditions by comparison of CD34 median intensity fluorescence (Figures 9A,B). Indeed, enhanced expansion of HSPC through a co-culture setting with MSC has also been observed in other studies. Beneficial impact compared to traditional liquid suspension has been described concerning cell expansion levels (da Silva et al., 2010; Fajardo-Orduña et al., 2017; Darvish et al., 2019), but also in what concerns the biological features of the cultured cells, for instances, contributing toward an enhanced migration capability of HSPC (Alakel et al., 2009; Perdomo-Arciniegas and Vernot, 2011). Overall, our evaluation of each expansion system after cytokine optimization has provided a more reliable and unbiased view over their genuine production capabilities of a potential expanded HSPC product.

To fully assess the viability of these systems as potential cell therapy platforms, the entire manufacturing process needs to be considered. Importantly, we have used cryopreserved UCB HSPC to mimic the UCB unit processing in current clinical trials, as these pioneering trials normally lay the groundwork for the manufacturing process of the respective approved product. With source cryopreservation being an important bioprocess step that can have an impact on the characteristics of the cell product (e.g., need for cell revitalization), disregarding it can also affect optimization applicability. Additionally, acquired process knowledge of cytokine interactions will also prove to be very useful in building such a manufacturing pipeline for an expanded HSPC product (Lipsitz et al., 2016). Determined regressions will provide critical information on expansion reaction and a degree of predictability if unavoidable changes in cytokine concentration should happen during production. However, expansion yield by itself is not the only priority in cell therapy development and an overall balance among operational parameters is needed (de Fuzeta et al., 2019). Although CS_HSPC/MSC was shown to produce a higher number of expanded HSPC with superior quality measures necessary for HCT, it also holds a higher level of complexity as a culture system. Normally, a trade-off between complexity and feasibility has existed in the manufacturing of cell therapies, hindering their translation (Dodson and Levine, 2015). In this case, the presence of a feeder layer in the expansion system will require add-ons or modifications to its manufacturing process when compared to the simpler CS_HSPC. An additional upstream source collection and isolation procedure for MSC will be needed, while downstream units will have to be able to separate MSC from expanded HSPC to assure end product purity. Also, preparation of MSC feeder layers inevitably increases the total culture duration and requires culture formats compatible with adherent cell culture. All these issues, which might prove challenging or costly, need to be considered and counterbalanced with the performance increase shown by CS_HSPC/MSC in product quantity and quality. Therefore, bioprocessing studies with economic modeling must accompany this co-culture system to determine if this more complex platform is worthwhile (Csaszar et al., 2013; Chilima et al., 2018; Mizukami et al., 2018; de Pinto et al., 2019).

Strategies similar to our experimental design should become widespread, as they represent a statistically sound and efficient way to reach optimal experimental conditions (Lipsitz et al., 2016; Toms et al., 2017). The methodology applied in our study, targeting the use of cytokines for *ex vivo* HSPC expansion, can be translated to other culture parameters and applications. Stem cell fate studies (self-renewal vs. differentiation) (Barbosa et al., 2012; Dias et al., 2019), as well as biomaterial development for tissue engineering (Levin et al., 2018), are fields that are centered on continuous improvement and optimization of experimental conditions in order to reach a defined differentiated cell type or scaffold, respectively. Filled with possible optimization parameters (e.g., differentiation media, oxygen tension, cell aggregate size, scaffold porosity, stirring speed in bioreactor systems, etc.), studies benefit immensely by using experimental design as they avoid unnecessary iterations of dose-response experiments, reduce reagent and material costs and become more time-efficient (Levin et al., 2018; Branco et al., 2019).

With our study, we had aimed at addressing three different goals. Initially, with expansion strategies reaching or advancing through the clinical trial pipeline, we recognized a window of opportunity where performed optimizations could be implemented without losing their applicability and sharing the same fate as previous studies. We were successful in optimizing each of the studied expansion systems, leading to improved expanded HSPC products with higher expansion yields while potentially maintaining the quality necessary for expanded HSPC to produce their expected therapeutic value (i.e., reconstitute the entire hematopoietic system), characterized herein by their CD34^+^ expression and multilineage-potential. Thus, we have produced enduring optimizations that directly influence clinically important HSPC expansion platforms or may even guide novel HSPC expansion strategies in the future. Secondly, as these strategies are simultaneously progressing toward regulatory approval, we had also ambitioned establishing optimization as a tool to perform a rational and comprehensive evaluation between different approaches. By doing so, differences between CS_HSPC and CS_HSPC/MSC were emphasized in an unbiased manner. Our study can influence decision-making and risk analysis of both systems as expansion platforms undergoing regulatory funneling to reach commercial approval. Finally, by increasing process knowledge on cytokine supplementation, we have contributed toward the implementation of an approved UCB-derived *ex vivo* expanded HSPC therapy concerning its manufacturing and production process. Moreover, we have promoted the framework behind our study and its results to be used for other potential stem cell-based products outside its original scope, in terms of development, manufacturing and economic perspectives.

The optimization performed herein will allow further work to build on improved expansion platforms. Having ascertained the necessary cytokine requirements for CS_HSPC/MSC and CS_HSPC systems, we expect to explore the scalability of these platforms. With a seeding density of 30 000 cells/mL, the validation of larger expansion volumes with optimized culture conditions will provide an opportunity to reach clinically relevant cell numbers and contribute toward the establishment of expanded HSPC as a clinically viable cell therapy. Also, recognizing the limitations of relying on immunophenotypic characterization as a predictor of therapeutic potential for expanded HSPC performed in clinical trials (e.g., expression of CD34), we anticipate an increased relevance of an omics-approach. With more thorough techniques in characterizing cell function, we anticipate that a possible cellular signature linked to therapeutic action in expanded HSPC may be unraveled, allowing the establishment of robust potency and functional assays for such cell-based products which are currently lacking (Kirouac and Zandstra, 2008).

## Data Availability Statement

All datasets generated for this study are included in the article/Supplementary Material.

## Ethics Statement

UCB units and BM samples were obtained from healthy donors, upon informed consent, with the approval of the ethics committee of Hospital São Francisco Xavier, Centro Hospitalar de Lisboa Ocidental and of Instituto Português de Oncologia Francisco Gentil, Lisboa, Portugal, respectively.

## Author Contributions

AB, AF-P, and CLdS designed the research study and wrote the manuscript. AB conducted the experiments and modeled the data. SB and JM-S conducted the experiments. CL performed the umbilical cord blood collection, as well as donor screening and evaluation of donor suitability for our study. All authors critically revised and approved the final manuscript.

## Conflict of Interest

The authors declare that the research was conducted in the absence of any commercial or financial relationships that could be construed as a potential conflict of interest.

## Glossary

UCB: umbilical cord blood
HSPC: hematopoietic stem/progenitor cells
CS_HSPC: HSPC culture system
BM MSC: bone marrow derived mesenchymal stromal cells
CS_HSPC/MSC: co-culture system with BM MSC
SCF: stem cell factor
Flt-3L: fms-like tyrosine kinase 3 ligand
TPO: thrombopoietin
FI: fold increase
FI TNC: fold increase in total nucleated cells
BFU-E: erythroid burst-forming unit
CFU-GM: colony-forming unit granulocyte-monocyte
CFU-Mix: multilineage colony-forming unit
HCT: hematopoietic cell transplantation
BM: bone marrow
GVHD: graft vs. host disease
HLA: human leukocyte antigen
G-CSF: granulocyte colony-stimulating factor
IL-3: interleukin-3
IL-6: Interleukin-6
GMP: good manufacturing practices
MNC: mononuclear cells
USA: United States of America
PBS: phosphate buffered saline
EDTA: ethylenediamine tetraacetic acid
DMEM: Dulbecco’s Modified Eagle’s medium
FBS: fetal bovine serum
SCERG: Stem Cell Engineering Research Group
bFGF: basic fibroblast growth factor
CAFC: cobblestone area forming-cells
RT: room temperature
CCF: face-centered central composite
RMSE: root mean squared error
SEM: standard error of the mean
CV: coefficient of variation
R^2^: coefficient of determination
MFI: median fluorescence intensity
QbD: quality by design
α -MEM: Minimum Essential Medium Eagle-Alpha Modification
IMDM: Iscove’s Modified Dulbecco’s Medium.
