# Supplementary material for: Tailored Cytokine Optimization for ex vivo Culture Platforms Targeting the Expansion of Human Hematopoietic Stem/Progenitor Cells
Source: Front Bioeng Biotechnol. 2020 Sep 25;8:573282. doi: 10.3389/fbioe.2020.573282 (PMC7729524; doi:10.3389/fbioe.2020.573282)
Supplement: Supplementary file 1 [file Table_1.DOCX]

Supplementary Material

Figure S1 - Preparation and polishing of experimental data with assessment of regression quality for FI CD34^+^ cells for both expansion systems, HSPC suspension culture (CS_HSPC) and HSPC co-cultured with BM MSC (CS_HSPC/MSC). **(A)** Data from cells retrieved from every UCB donor was normalized revealing coinciding reaction patterns, highlighting variability exclusively due to different cytokine combinations. **(B)** Outlier screening was performed through Z-score determination. Data points with absolute score values higher than 3 were labelled outliers and were consequently removed from their data set before proceeding to the regression determination. **(C)** After regression determination, experimental data points were compared with calculated regression. **(D)** Deviations between data points and regressions were visualized. Norm – normalized.

Figure S2 - Preparation and polishing of experimental data with assessment of regression quality for FI CFU-GM for both expansion systems, HSPC suspension culture (CS_HSPC) and HSPC co-cultured with BM MSC (CS_HSPC/MSC). **(A)** Data from cells retrieved from every UCB donor was normalized revealing coinciding reaction patterns, highlighting variability exclusively due to different cytokine combinations. **(B)** Outlier screening was performed through Z-score determination. Data points with absolute score values higher than 3 were labelled outliers and were consequently removed from their data set before proceeding to the regression determination. **(C)** After regression determination, experimental data points were compared with calculated regression. **(D)** Deviations between data points and regressions were visualized. Norm – normalized.

Figure S3 - Preparation and polishing of experimental data with assessment of regression quality for FI CFU-Mix for both expansion systems, HSPC suspension culture (CS_HSPC) and HSPC co-cultured with BM MSC (CS_HSPC/MSC). **(A)** Data from cells retrieved from every UCB donor was normalized revealing coinciding reaction patterns, highlighting variability exclusively due to different cytokine combinations. **(B)** Outlier screening was performed through Z-score determination. Data points with absolute score values higher than 3 were labelled outliers and were consequently removed from their data set before proceeding to the regression determination. **(C)** After regression determination, experimental data points were compared with calculated regression. **(D)** Deviations between data points and regressions were visualized. Norm – normalized.

Table S1 – Number of CFU populations per seeded cells for HSPC suspension culture (CS_HSPC) and HSPC co-cultured with BM MSC (CS_HSPC/MSC) of the optimization study and its validation. SEM – Standard error of the mean

| **Optimization** | | | | | | | | | | | | |
| --- | --- | --- | --- | --- | --- | --- | --- | --- | --- | --- | --- | --- |
|  | CS_HSCP/MSC | | | | | | CS_HSPC | | | | | |
|  | BFU-E | | CFU-GM | | CFU-Mix | | BFU-E | | CFU-GM | | CFU-Mix | |
| Comb. | Mean | SEM | Mean | SEM | Mean | SEM | Mean | SEM | Mean | SEM | Mean | SEM |
| 1 | 9.5x10^-4^ | 5.8x10^-4^ | 6.8x10^-2^ | 1.8x10^-2^ | 1.5x10^-2^ | 4.1x10^-3^ | 5.7x10^-4^ | 5.7x10^-4^ | 4.4x10^-2^ | 2.3x10^-2^ | 2.6x10^-2^ | 1.5x10^-2^ |
| 2 | 5.7x10^-4^ | 3.3x10^-4^ | 6.6x10^-2^ | 1.9x10^-2^ | 1.5x10^-2^ | 5.5x10^-3^ | 2.1x10^-3^ | 1.4x10^-3^ | 6.3x10^-2^ | 1.6x10^-2^ | 2.3x10^-2^ | 2.2x10^-3^ |
| 3 | 1.4x10^-3^ | 8.7x10^-4^ | 6.0x10^-2^ | 1.4x10^-2^ | 3.1x10^-2^ | 3.1x10^-3^ | 3.8x10^-4^ | 9.5x10^-5^ | 4.4x10^-2^ | 1.8x10^-2^ | 4.2x10^-2^ | 8.1x10^-3^ |
| 4 | 3.5x10^-3^ | 2.0x10^-3^ | 7.2x10^-2^ | 2.4x10^-2^ | 1.4x10^-2^ | 4.9x10^-3^ | 1.8x10^-3^ | 9.7x10^-4^ | 4.9x10^-2^ | 1.8x10^-2^ | 2.6x10^-2^ | 4.6x10^-3^ |
| 5 | 1.0x10^-3^ | 6.7x10^-4^ | 5.7x10^-2^ | 1.5x10^-2^ | 1.9x10^-2^ | 8.3x10^-3^ | 3.8x10^-4^ | 3.8x10^-4^ | 5.0x10^-2^ | 1.9x10^-2^ | 3.9x10^-2^ | 7.2x10^-3^ |
| 6 | 1.0x10^-3^ | 5.3x10^-4^ | 6.3x10^-2^ | 1.7x10^-2^ | 8.6x10^-3^ | 3.2x10^-3^ | 2.9x10^-4^ | 0 | 6.8x10^-2^ | 2.5x10^-2^ | 1.5x10^-2^ | 6.2x10^-3^ |
| 7 | 1.1x10^-3^ | 8.7x10^-4^ | 4.3x10^-2^ | 8.4x10^-3^ | 4.1x10^-2^ | 9.1x10^-4^ | 4.1x10^-3^ | 3.0x10^-3^ | 2.5x10^-2^ | 1.1x10^-2^ | 3.7x10^-2^ | 8.2x10^-3^ |
| 8 | 3.8x10^-4^ | 2.5x10^-4^ | 4.6x10^-2^ | 1.7x10^-2^ | 2.0x10^-2^ | 4.6x10^-3^ | 2.0x10^-3^ | 1.7x10^-3^ | 1.8x10^-2^ | 8.7x10^-3^ | 9.3x10^-3^ | 3.3x10^-3^ |
| 9 | 8.6x10^-4^ | 4.4x10^-4^ | 6.2x10^-2^ | 1.8x10^-2^ | 2.1x10^-2^ | 3.2x10^-3^ | 2.2x10^-3^ | 5.0x10^-4^ | 5.3x10^-2^ | 2.0x10^-2^ | 4.6x10^-2^ | 8.9x10^-3^ |
| 10 | 6.7x10^-4^ | 6.7x10^-4^ | 5.6x10^-2^ | 1.7x10^-2^ | 1.7x10^-2^ | 3.5x10^-3^ | 4.8x10^-3^ | 2.9x10^-3^ | 5.2x10^-2^ | 2.2x10^-2^ | 4.0x10^-2^ | 1.3x10^-2^ |
| 11 | 2.0x10^-3^ | 1.4x10^-3^ | 5.7x10^-2^ | 1.5x10^-2^ | 1.7x10^-2^ | 2.0x10^-3^ | 1.0x10^-3^ | 6.7x10^-4^ | 5.1x10^-2^ | 1.7x10^-2^ | 4.1x10^-2^ | 8.5x10^-3^ |
| 12 | 1.5x10^-3^ | 7.8x10^-4^ | 5.1x10^-2^ | 1.9x10^-2^ | 1.6x10^-2^ | 4.4x10^-3^ | 9.5x10^-5^ | 9.5x10^-5^ | 5.1x10^-2^ | 2.0x10^-2^ | 4.3x10^-2^ | 7.3x10^-3^ |
| 13 | 1.4x10^-3^ | 3.3x10^-4^ | 6.7x10^-2^ | 1.7x10^-2^ | 1.4x10^-2^ | 4.2x10^-3^ | 2.6x10^-3^ | 1.6x10^-3^ | 6.3x10^-2^ | 2.4x10^-2^ | 2.1x10^-2^ | 3.5x10^-3^ |
| 14 | 1.0x10^-3^ | 5.8x10^-4^ | 6.0x10^-2^ | 1.1x10^-2^ | 2.2x10^-2^ | 4.4x10^-3^ | 4.8x10^-4^ | 4.8x10^-4^ | 4.7x10^-2^ | 1.7x10^-2^ | 3.9x10^-2^ | 4.3x10^-3^ |
| 15 | 1.3x10^-3^ | 3.4x10^-4^ | 6.1x10^-2^ | 1.9x10^-2^ | 1.7x10^-2^ | 5.8x10^-3^ | 1.9x10^-4^ | 1.9x10^-4^ | 4.5x10^-2^ | 1.7x10^-2^ | 4.0x10^-2^ | 3.7x10^-3^ |
| 16 | 1.0x10^-3^ | 5.8x10^-4^ | 6.2x10^-2^ | 1.6x10^-2^ | 1.4x10^-2^ | 5.1x10^-3^ | 1.9x10^-3^ | 1.0x10^-3^ | 5.6x10^-2^ | 1.6x10^-2^ | 4.2x10^-2^ | 8.8x10^-3^ |
| 17 | 9.5x10^-4^ | 4.2x10^-4^ | 6.0x10^-2^ | 1.5x10^-2^ | 2.6x10^-2^ | 8.8x10^-3^ | 2.9x10^-4^ | 1.6x10^-4^ | 3.9x10^-2^ | 1.3x10^-2^ | 4.7x10^-2^ | 9.8x10^-3^ |
|  | | | | | | | | | | | | |
| Validation | | | | | | | | | | | | |
|  | CS_HSCP/MSC | | | | | | CS_HSPC | | | | | |
|  | BFU-E | | CFU-GM | | CFU-Mix | | BFU-E | | CFU-GM | | CFU-Mix | |
| Cocktail | Mean | SEM | Mean | SEM | Mean | SEM | Mean | SEM | Mean | SEM | Mean | SEM |
| Z9 | 1.7x10^-3^ | 1.1x10^-2^ | 6.5x10^-2^ | 1.1x10^-2^ | 2.6x10^-2^ | 6.4x10^-3^ | 2.6x10^-3^ | 1.7x10^-3^ | 5.3x10^-2^ | 1.3x10^-2^ | 3.7x10^-2^ | 1.6x10^-2^ |
| AB20 | 3.3x10^-3^ | 4.6x10^-3^ | 5.4x10^-2^ | 4.6x10^-3^ | 4.8x10^-2^ | 1.6x10^-2^ | 8.6x10^-4^ | 5.7x10^-4^ | 3.9x10^-2^ | 1.2x10^-2^ | 5.6x10^-2^ | 8.6x10^-4^ |

Table S2 – List of reagents and consumables and their respective product references.

| Reagent | Source | Identifier |
| --- | --- | --- |
| Ficoll-Paque Premium | GE Healthcare | 17544203 |
| PBS | Thermo Fisher Scientific | 21600-044 |
| EDTA | Sigma-Aldrich | 03690-100mL |
| Ammonium Chloride (NH_4_Cl) | Sigma-Aldrich | 254134-25G |
| Recovery™ Cell Culture Freezing Medium | Thermo Fisher Scientific | 12648010 |
| FBS | Thermo Fisher Scientific | 10270106 |
| DMSO | Thermo Fisher Scientific | D8418-100ML |
| Recovery Medium | Thermo Fisher Scientific | 12648-010 |
| DMEM | Thermo Fisher Scientific | 31600-091 |
| Antibiotic/Antimycotic (100X) | Thermo Fisher Scientific | 15240-062 |
| DNase I | Sigma-Aldrich | DN25-100MG |
| Human CD34 MicroBead Kit UltraPure | Miltenyi Biotec | 130-100-453 |
| FBS MSC-certified | Thermo Fisher Scientific | 12662-029 |
| Mitomycin C | Sigma-Aldrich | M4287-2MG |
| StemSpan SFEM II | STEMCELL Technologies | 09655 |
| SCF | PeproTech | AF-HHSC3 |
| Flt3L | PeproTech | AF-HHSC3 |
| TPO | PeproTech | AF-HHSC3 |
| bFGF | PeproTech | AF-100-18B |
| Trypan Blue | Thermo Fisher Scientific | 15250-061 |
| MethoCult™ Classic | STEMCELL Technologies | 04434 |
| MyeloCult™ | STEMCELL Technologies | 05150 |
| Hydrocortisone | STEMCELL Technologies | 07904 |
| LIVE/DEAD Fixable Far Red Dead Cell Stain Kit | Thermo Fisher Scientific | L34974 |
| Anti-Human CD34 FITC | BioLegend | 343504 |
| Anti-Human CD34 PE | BioLegend | 343506 |
| Anti-Human CD34 PerCP-Cy5.5 | BD Biosciences | 347222 |
| Anti-Human CD90 PE | BioLegend | 328110 |
| Formaldehyde | Sigma-Aldrich | 158127 |
